# Comparative Genomic Analysis and In Vivo Modeling of *Streptococcus pneumoniae* ST3081 and ST618 Isolates Reveal Key Genetic and Phenotypic Differences Contributing to Clonal Replacement of Serotype 1 in The Gambia

**DOI:** 10.1093/infdis/jix472

**Published:** 2017-09-14

**Authors:** Laura Bricio-Moreno, Chinelo Ebruke, Chrispin Chaguza, Jennifer Cornick, Brenda Kwambana-Adams, Marie Yang, Grant Mackenzie, Brendan W Wren, Dean Everett, Martin Antonio, Aras Kadioglu

**Affiliations:** 1 Department of Clinical Immunology, Microbiology and Immunology, Institute of Infection and Global Health, University of Liverpool; 2 Faculty of Infectious and Tropical Diseases, London School of Hygiene & Tropical Medicine; 3 Microbiology and Infection Unit, Warwick Medical School, University of Warwick, Coventry, United Kingdom; 4 Vaccines and Immunity Theme, Medical Research Council Unit, Banjul, The Gambia; 5 Malawi-Liverpool-Wellcome Trust Clinical Research Programme, Queen Elizabeth Central Hospital, Blantyre, Malawi

**Keywords:** Pneumococcus, clonal replacement, infection models, pathogenesis

## Abstract

*Streptococcus pneumoniae* serotype 1 is one of the leading causes of invasive pneumococcal disease (IPD) in West Africa, with ST618 being the dominant cause of IPD in The Gambia. Recently however, a rare example of clonal replacement was observed, where the ST3081 clone of serotype 1 replaced the predominant ST618 clone as the main cause of IPD. In the current study, we sought to find the reasons for this unusual replacement event. Using whole-genome sequence analysis and clinically relevant models of in vivo infection, we identified distinct genetic and phenotypic characteristics of the emerging ST3081 clone. We show that ST3081 is significantly more virulent than ST618 in models of invasive pneumonia, and is carried at higher densities than ST618 during nasopharyngeal carriage. We also observe sequence type–specific accessory genes and a unique sequence type–specific fixed mutation in the pneumococcal toxin pneumolysin, which is associated with increased hemolytic activity in ST3081 and may contribute to increased virulence in this clone. Our study provides evidence that, within the same serotype 1 clonal complex, biological properties differ significantly from one clone to another in terms of virulence and host invasiveness, and that these differences may be the result of key genetic differences within the genome.

## Teaser

In this study we provide important evidence to show that changes in the epidemiology of pneumococcal serotype 1 sequence types in The Gambia may be a direct consequence of differences in virulence and increased ability to colonize hosts over time.

To date, nearly 100 distinct serotypes of *Streptococcus pneumoniae* (pneumococcus) have been reported [1–7]. In the West African subregion as well as globally, pneumococcal serotype 1 is one of the leading causes of invasive pneumococcal disease (IPD) [8–14]. This serotype is known to cause disease outbreaks, particularly in the African meningitis belt, where the serotype 1 ST217 hypervirulent clonal complex has been predominant [15–20]. An epidemiological review of meningitis cases in the African meningitis belt revealed that serotype 1 was as important as *Neisseria meningitidis* in its contribution to the incidence and mortality caused by bacterial meningitis [10]. Despite its strong association with invasive disease, serotype 1 is rarely detected during nasopharyngeal carriage. In The Gambia, serotype 1 is one of the most common causes of IPD responsible for up to 20% of cases, yet is identified in only 0.5% of healthy carriers [8, 18, 21, 22]. Compared with other pneumococcal serotypes, serotype 1 has a high odds ratio to cause IPD after colonization [23] and is rarely associated with antibiotic nonsusceptibility [8, 15, 18]. It has previously been demonstrated that long-range transmission of serotype 1 is rare, which leads to stable circulating clones within geographic regions [24]. Newly imported clones have little impact on the epidemiology of serotype 1, but we recently reported clonal replacement in serotype 1 in The Gambia where the ST3081 clone replaced the predominant ST618 as the main cause of IPD [24]. The reasons for this were unknown, however.

In the current study, we investigated whether this clonal replacement event was a consequence of the presence of virulence associated genes in the accessory genome of ST3081, which were absent in the accessory genome of ST618. To this end, we describe the population structure of invasive *S. pneumoniae* serotype 1 isolates collected in The Gambia over a 20-year period between January 1995 and December 2014, and compare the genomic profiles and in vivo virulence, colonization and invasiveness of these 2 important evolving clones.

## MATERIALS AND METHODS

### Epidemiological Study Design and Collection of Isolates

The study collection included all *S. pneumoniae* serotype 1 isolates sent to the World Health Organization Regional Reference Laboratory for invasive bacterial diseases in the Medical Research Council (MRC) Unit of The Gambia, between January 2004 and December 2014 as part of the routine hospital surveillance for IPD in The Gambia. Samples originated from the Western (urban), Central River (rural), and Upper River (rural) regions of The Gambia (Supplementary Table S1). We also included a previous study collection (1995–2003) of 127 invasive Gambian isolates [18] and compared these with the 190 isolates obtained from 2004 to 2014 (Supplementary Table S1). Sites of isolation included blood, cerebrospinal fluid, lung aspirates, pleural aspirates, or a combination of sites. Twenty-one pneumococcal carriage isolates (Supplementary Table S1) were obtained from a collection of serotype 1 nasopharyngeal isolates from healthy individuals in The Gambia, as described elsewhere [22]. When >1 isolate was obtained during an illness episode, for example from blood and cerebrospinal fluid, only a single isolate was included in the study collection, except when the isolates differed in multilocus sequence type (MLST) or antibiotic susceptibility profile. Isolates were stored in 15% glycerol stocks before study. Isolation of *S. pneumoniae* and serotyping by latex agglutination was done as described elsewhere [8]. Serotype 1 isolates were confirmed by molecular serotyping, also as described elsewhere [25].

### Hemolytic Activity Assay

The hemolytic activity of the invasive ST3081 (6308 7#2) and ST618 (6309 7#1) isolates was determined using a modified version of a previously published method [26]. An extra 6 isolates per sequence type (ST) were tested for confirmation of the results. A single colony of each isolate was grown in brain heart infusion to a late exponential phase and lysed with 0.1% sodium deoxycholate. Approximately 85 µg of total protein was serially diluted and incubated with a 4% suspension of sheep red blood cells (Oxoid). The suspension was centrifuged after a 30-minute incubation at 37°C. The supernatants’ optical density at 540 nm was measured to determine the hemolytic activity of each isolate.

### In Vivo Models of Nasopharyngeal Carriage and Invasive Pneumonia

All in vivo experiments were done at the University of Liverpool under the UK Animals (Scientific Procedures) Act 1986 guidelines. The protocols were approved by the UK Home Office and by the University of Liverpool Animal Welfare and Ethics Committee. All animals were regularly monitored during the duration of the experiments and humanely culled at predetermined time points or when they became lethargic. Outbred MF1 female mice, 6–8 weeks old (Charles River) were used in the study. The animals were left to acclimatize for a minimum of 7 days before experimentation.

The invasive pneumonia experiments were performed using the ST618 (6309 7#1) and ST3081 (6308 7#2) invasive isolates while the nasopharyngeal carriage experiments were performed using the ST618 (6308 8#18) and ST3081 (6308 8#10) carriage isolates. The strains used were representative isolates of each ST. Liquid bacterial stocks were used for short term usage by growing a sweep of colonies into 20% serum brain heart infusion for approximately 12 hours at 37°C. Stocks were then stored at –80°C and viable counts determined using the method described by Miles et al [27]. When needed, the bacterial stocks were thawed, washed with sterile phosphate-buffered saline (PBS), and adjusted to the appropriate density.

Mice were anesthetized with a mixture of oxygen and isoflurane (Abbott) and intranasally infected with 10^6^ colony-forming units (CFUs) in a 50-µL volume of sterile PBS for the invasive pneumonia model or with 10^5^ CFUs in a 10-µL volume of sterile PBS for the nasopharyngeal carriage model, as described elsewhere [28, 29]. For the nasopharyngeal cocarriage model, mice were intranasally infected with 10 µL of PBS containing 5 × 10^4^ CFUs of ST618 and 5 × 10^4^ CFUs of ST3081 pneumococci. Blood, lung, brain, and nasopharyngeal tissues were collected to determine CFU counts. Blood agar plates containing 1 µg/mL of gentamicin were used to determine the total number of viable pneumococci; however, plates containing 200 µg/mL of streptomycin were used to determine the number of ST3081 viable counts in the cocarriage experiment. In that case the CFU counts for ST618 were determined by subtracting the number of colonies in the streptomycin plates from the number of colonies in the gentamicin plates.

### Comparative Genomic Analysis

Seventy-four isolates comprising 53 invasive and 21 carriage isolates, ST3081 (n = 38) and ST618 (n = 36), were sequenced at the Wellcome Trust Sanger Institute using the Illumina Genome Analyzer II (Illumina) (Supplementary Table S2). We used SMALT software (version 0.7.4; http://www.sanger.ac.uk/resources/software/smalt/) to map the Illumina short read sequences against the serotype 1 reference whole-genome sequence SPN1041 (Genbank No. CACE00000000) to generate the consensus pseudochromosomes for all of the study isolates, which were combined and aligned to generate a whole-genome alignment. All nucleotide substitutions were detected using Gubbins software (version 1.1.1) [30] and removed to avoid conflicting evolutionary signals when constructing phylogenetic tree of the STs. A phylogenetic tree was constructed using RAxML software (version 7.0.4) [31]. The following parameters were used for RAxML: generalized time-reversible model with gamma heterogeneity among nucleotide sites and 100 bootstrap replicates. The phylogenetic trees were visualized using FigTree software (http://tree.bio.ed.ac.uk/software/figtree/) and BioPython scripts [32]. Genome assembly was performed using Velvet (version 1.2.09) [33] and SSPACE Basic (version 2.0) [34] software.

We calculated the pairwise nucleotide differences using BioPython scripts [32]. Additional evolution parameters; number of single-nucleotide polymorphism (SNP) sites, number of SNPs per nucleotide, observed (π) and expected (Θ) nucleotide sequence diversity and Tajima’s *D,* which is the scaled difference between π and Θ, were calculated using MEGA software (version 6.0) [35]. To investigate the temporal evolution patterns within each ST and to estimate the times to the most recent common ancestors or potential emergence of the STs, we used Path-O-gen software v1.4 (http://tree.bio.ed.ac.uk/software/pathogen/).

### Data Analysis

To determine the geographic relationship of Gambian serotype 1 clones to those found in other regions of the world, we performed cluster analysis of allelic profiles of serotype 1 isolates fro m this study and from the MLST database, using geoBURST software v1.2.1 (http://goeBURST.phyloviz.net). The statistical analysis related to the in vivo mouse experiments was carried out using the GraphPad Prism statistical package (version 5; GraphPad Software). Statistical significance was categorized as ^*^P < .05, **P < .01,***P < .005, or ****P < .001.

## RESULTS

### Multilocus Sequence Typing and Molecular Epidemiology

**Figure 1.**
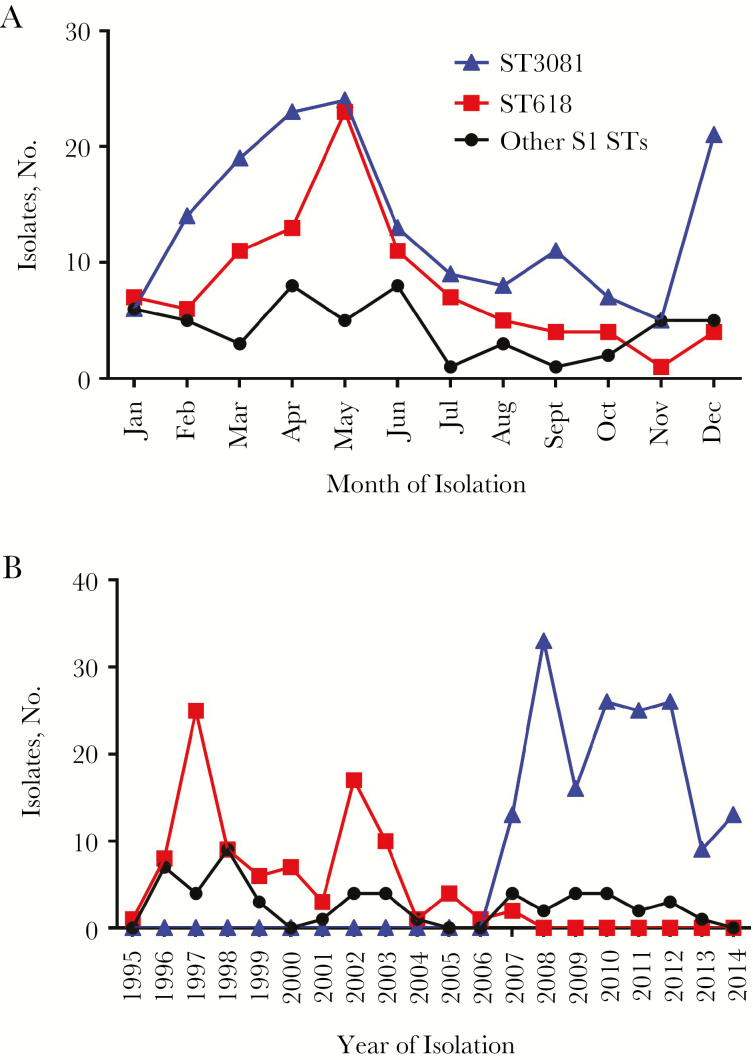
*A,* Seasonality in the monthly distribution of invasive serotype 1 sequence types (STs; ST618, ST3081, and other S1 STs) in Gambia between January 1995 and December 2014. *B,* Yearly multilocus ST data of Gambian serotype 1 isolates from 1995 to 2014.

The majority of pneumococcal serotype 1 isolates were obtained between December and June (hot dry season) with peaks at the beginning and at the end of the season (Figure 1A). ST618 was the predominant ST until 2007, when it was replaced by ST3081 (Figure 1B). Within 1 year, ST618 was totally replaced by ST3081.

Using geoBURST analysis, we showed the existence of a geographic clustering in the Gambian STs compared with STs isolated from other parts of Africa, from Asia, and from around the world (Supplementary Figure S1). Whereas both ST3081 and ST618 clustered with clones from Africa and Asia, ST618 was positioned between Africa/Asia and the other parts of the world.

### In Vivo Characterization of ST618 and ST3081 in Models of Invasive Pneumonia

**Figure 2.**
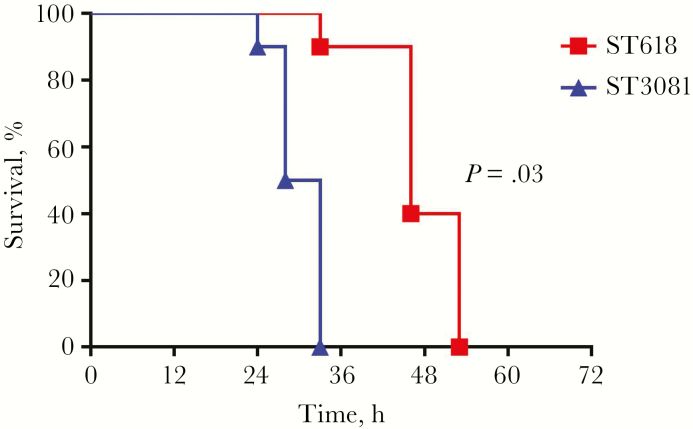
Survival of mice intranasally infected with 10^6^ colony-forming units of ST618 (isolate 6309 7#1) and ST3081 (isolate 6308 7#2; Ten mice infected with each isolate). *P* = .03 (log-rank [Mantel-Cox] test).

A mouse model of invasive pneumonia was used to compare the virulence of ST618 (6309 7#1) and ST3081 (6308 7#2) in vivo. Of the mice infected with the ST3081 isolate, 100% developed signs of lethargy approximately 24 hours after infection, and all succumbed to the infection within 33 hours . In contrast, only 10% of the mice infected with ST618 died within 33 hours; the remaining 90% died within 53 hours (Figure 2), a statistically significant difference (*P* = .03; log-rank [Mantel-Cox] test). 

**Figure 3.**
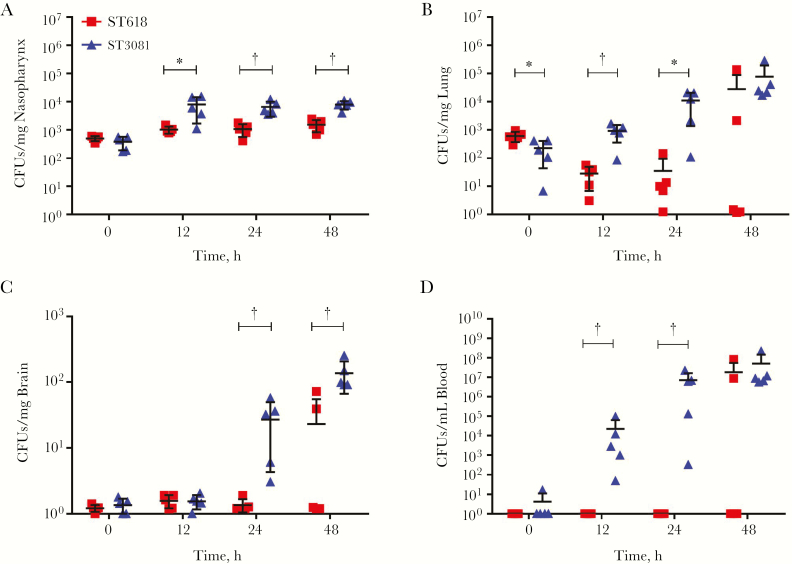
In vivo characterization of the invasive properties of ST3081 (isolate 6308 7#2; *triangles*) and ST618 (isolate 6309 7#1; *squares*). Colony-forming unit (CFU) counts are shown for nasopharyngeal (*A*), lung (*B*), and brain (*C*) tissue and for blood (*D*) during the 48 hours after infection (5 infected mice per time point per isolate). *P < .05; †*P* < .01 (2-way analysis of variance).

In time-pointed experiments over 48 hours, mice infected with ST3081 pneumococci had significantly higher bacterial loads in the nasopharynx at 12, 24, and 48 hours after infection (Figure 3A). Similarly, in the lungs, mice infected with ST3081 had significantly higher bacterial loads than the ST618-infected mice at 12 and 24 hours after infection, with increasing CFU counts at 48 hours (Figure 3B). In the brain, no significant differences in CFU counts were observed in the first 12 hours after infection, but by 24 and 48 hours after infection, mice infected with ST3081 had significantly higher bacterial loads in the brain. Interestingly, there were no detectable CFUs for ST618 at 24 hours, demonstrating a clear difference in systemic tissue invasiveness between the 2 isolates (Figure 3C). The same pattern was observed in the blood, where ST3081-infected mice had significantly higher bacterial CFU counts in blood by 12 hours after infection, reaching approximately 10^7^ CFUs/mL by 24 hours. Once again, ST618 was not detectable at either 12 or 24 hours after infection, indicating that this ST is less invasive during the first 24 hours of infection (Figure 3D). In all experiments, the starting infection inoculum at time 0 was the same.

### In Vivo Characterization of ST3081 and ST618 in Models of Nasopharyngeal Carriage

**Figure 4.**
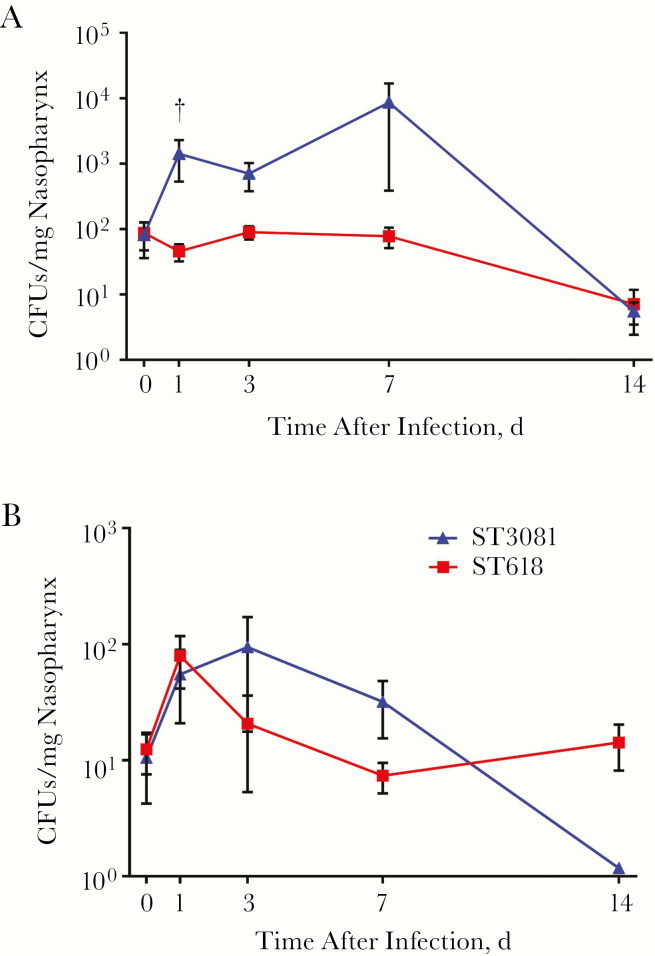
Characterization of carriage dynamics of ST3081 and ST618 in mice after single inoculation. *A,* Nasopharyngeal colony-forming unit (CFU) counts for ST618 (isolate 6308 8#18) or ST3081 (isolate 6308 8#10) during the 14 days after infection (5–10 mice infected with each isolate). *B,* Nasopharyngeal CFU counts in mice infected simultaneously with the ST618 and ST3081 isolates (8–10 mice infected). †*P* < .01 (Mann-Whitney *U* test).

To determine whether ST3081 and ST618 differ in their long-term carriage properties, we infected mice with a single inoculum of each isolate, separately, and monitored density and duration of carriage during the 14 days after infection (Figure 4A). ST618 carried in the nasopharynx stably over 7 days, with a gradual but nonsignificant decline in CFU counts by day 14. On the other hand, CFU counts for ST3081 increased significantly during the first 7 days after infection. This sharp increase in bacterial density peaked at day 7, however, with CFU counts dropping significantly by day 14 compared with the previous 3 time-points. Overall, ST3081 was carried at higher CFU counts during the first 7 days, compared with ST618, although by day 14 carriage density was the same.

We next sought to assess whether ST3081 had a competitive advantage over ST618 to colonize the nasopharynx, by simultaneously coadministering mice with the 2 isolates and monitoring density and duration of carriage of each respective strain over 14 days. Interestingly, when both isolates were coadministered together, ST618 exhibited an increase in CFU counts within the first 24 hours, in contrast to its single-inoculum carriage state. However, this increase in bacterial density was reduced by day 3 and reached a steady state by day 7 with persistence of carriage maintained thereafter. In contrast, ST3081 showed a similar carriage profile to its single carriage state over the first 3 days after infection, although the peak CFU count was at day 3 (compared with day 7 in single-inoculum experiments). Density of carriage showed a steady decline in the cocarriage model over time; however, the bacterial density of ST3081 remained higher than that of ST618 between days 3 and 7 after infection (Figure 4B). These observations show that both STs follow similar carriage patterns during single colonization and cocolonization, with ST3081 able to be carried at a higher density than ST618.

### Comparative Genomic Analysis

**Figure 5.**
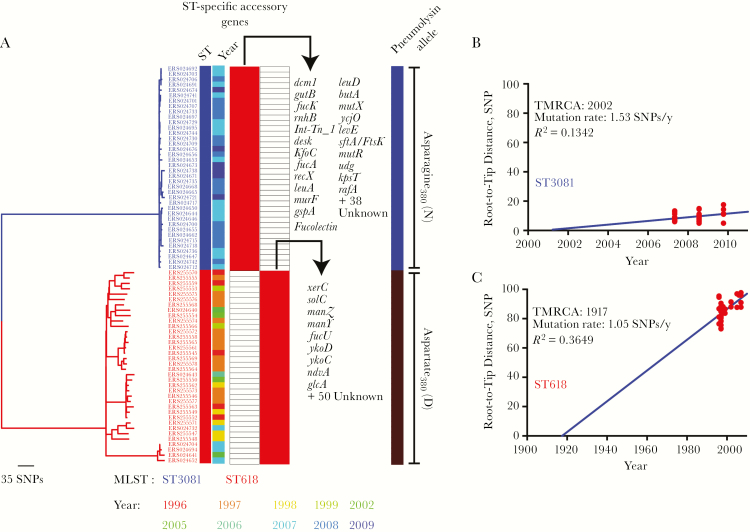
Evolution and emergence of the ST618 and ST3081 serotype 1 isolates in The Gambia. *A,* Distribution of accessory genes and mutations in the 380 position of the pneumolysin gene, specific to ST618 and ST3081 serotype isolates in Gambia. MLST, multilocus sequence type; SNPs, single-nucleotide polymorphisms; ST, sequence type. *B, C,* Nucleotide substitution (mutation) rates and potential years of emergence of the ST3081 (*B*) and ST618 (*C*) serotype 1 clones. TMRCA, time to the most common ancestor.

Phylogenetic analysis determined that the ST3081 and ST618 clones belonged to distinct monophyletic clades separated by long branches (Figure 5A). This suggests that ST3081, was not a descendant of ST618 but rather emerged as an independent clone from a recent introduction of a novel ST217 sublineage in The Gambia or as a local clonal expansion of a previously unreported clone. We performed a linear regression analysis of the ST-specific phylogenies to determine the correlation between the phylogenetic root-to-tip distances and the dates of isolation. The time to the most recent common ancestor for ST3081 was approximately 2002, which suggests recent introduction into The Gambia (Figure 5B), whereas ST618 emerged circa 1917 (Figure 5C). Both STs show nearly identical nucleotide substitution (mutation) rates, with about 1 SNP introduced annually per isolate through spontaneous mutations only rather than through genetic recombination.

**Figure 6.**
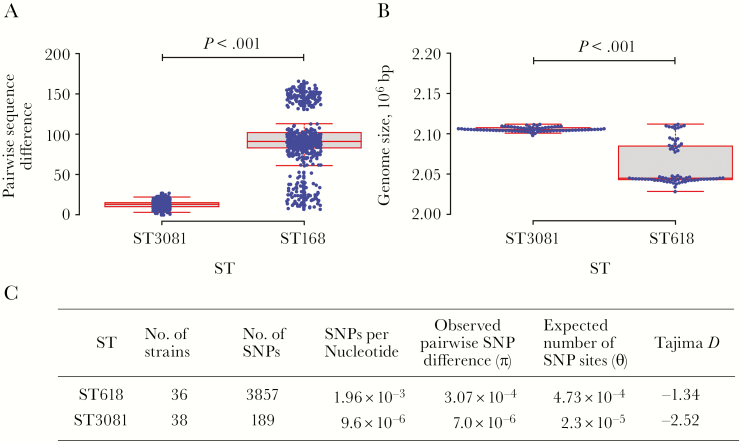
Nucleotide sequence diversity in ST618 and ST3081 serotype 1 clones in The Gambia. *A,* Number of single-nucleotide polymorphism (SNP) differences between 2 isolates in each sequence type (ST). *B,* Average genome size of the isolates in each ST; bp, base pairs *C,* Summary of the distribution of SNPs in each ST. The data were analyzed using a Student *t* test.

Significantly higher sequence diversity was observed in ST618 than in ST3081 (*P* < .001) (Figure 6A). In ST3081, 13 nucleotides distinguished the isolates, whereas this number increased to 93 nucleotides in ST618. On average, the ST618 isolates showed a smaller genome (*P* < .001) than ST3081, which may suggest genome degradation (Figure 6B); however, owing to the short sampling period it was not possible to accurately determine whether the genome degradation evident in ST618 occurred before the strain became endemic. We observed lower average pairwise sequence differences (π) than the expected number of sites with SNPs (Θ) in ST3081 than in ST618 (Figure 6C).

We performed a core genome analysis of the study strains to determine the number of core genes shared by both STs and accessory genes that were uniquely associated with a single ST. In this study we used an assembly based analysis, in contrast to the previously published analysis, which was done using a pangenomic mapping of reads approach [24]. Overall, both STs shared 1756 core genes, but a total of 120 genes (60 from each ST) were associated with a single ST (Figure 5A). In the previously published study it was found that ST3081 has accessory regions containing genes and operons implicated in pneumococcal virulence, such as *xerD* and the *ftsK/spoIIIE* operon, that are absent in ST618. 

**Figure 7.**
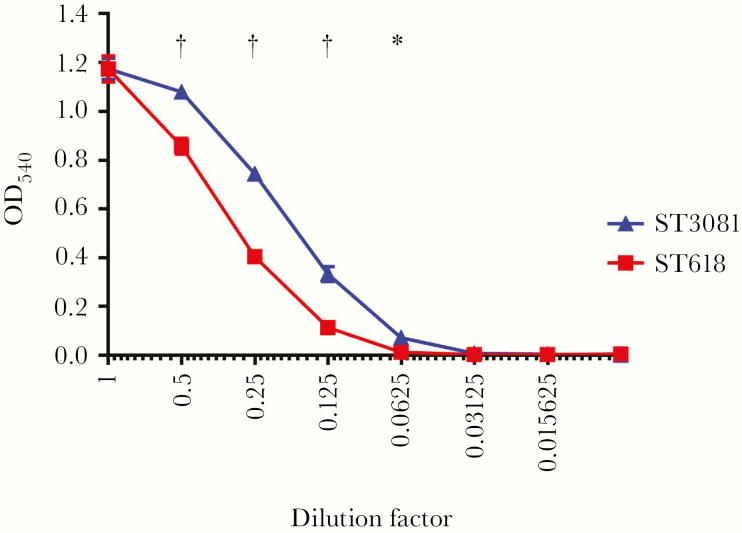
Hemolytic activity levels of the ST3081 (6308 7#2) and ST618 (6309 7#1) invasive isolates. *P < .01; †*P* < .001 (2-way analysis of variance). OD_540_, optical density at 540 nm.

In the current study, the assembly-based approach allowed us to identify an additional ST3081 unique gene, *murF*, which is involved in the biosynthesis of peptidoglycan. It has been shown in the past that *murF* impairment can lead to growth arrest and autolysis at high temperatures and at low environmental carbon dioxide levels [36]. Other uncharacterized ST3081 unique accessory genes could also provide a colonization advantage to ST3081 over ST618; however, the exact function of these remain unknown. In addition, 1 amino acid change in the pneumolysin protein which was unique to each ST was observed. At position 380, all ST3081 isolates contained an asparagine (N) whereas all ST618 consisted of an aspartic acid (D) (Figure 5A). The presence of asparagine at position 380 in pneumolysin protein has previously been associated with increased hemolytic activity in serotypes that cause outbreaks, suggesting that ST3081 pneumolysin may have higher hemolytic capacity than ST618 [26]. This was confirmed by our own hemolytic assays on the representative strains used in this study (Figure 7) as well as on another 12 strains (6 strains per ST) tested (Supplementary Table S3).

## DISCUSSION

Identification of the bacterial and host factors that control and regulate pneumococcal colonization of the nasopharyngeal niche is critical to our understanding of transmission events and invasive disease. Neill et al [37] have previously highlighted the key role of the pneumococcal toxin pneumolysin and the T-regulatory cell–transforming growth factor β axis in controlling establishment and duration of nasopharyngeal carriage [37]. Here, we report on the bacterial genetic and phenotypic factors which drive pneumococcal serotype 1 replacement in The Gambia, focusing on the capacity of 2 clonal STs to establish colonization in the nasopharynx and cause invasive disease.

Using newly acquired genome sequence data, we determined the population dynamics of the 2 dominant STs of pneumococcal serotype 1 in The Gambia over a period of 2 decades (Figure 1B), covering the introduction of 7-valent and 13-valent pneumococcal conjugate vaccine in 2009 and 2011, respectively. We show that the emergence of ST3081 was observed before the introduction of 7-valent pneumococcal conjugate vaccine in 2009, suggesting the absence of any vaccine-driven selective pressure [38, 39]; and that ST3081 continues to persist as the dominant clone since its first detection in 2007, suggesting that factors other than vaccine introduction may have caused this clonal replacement. We document that the newly dominant ST3081 clone occurred in both invasive disease and carriage isolates, and across both rural and urban settings in The Gambia, (Supplementary Table S1 and [22]). The same ST3081 clone was reported in neighboring Senegal [24], suggesting a possible local migration of the strain within the population. We determined that ST3081 shared similar characteristics with ST618, including being seasonal, occurring mostly during the dry season and declining in the rainy season in a fashion similar to meningococcus, a pathogen known to have a high propensity to cause epidemic outbreaks.

The current literature describes nasopharyngeal colonization as a dynamic process with turnover of new strains or species [37, 40]. Using mouse infection models, we sought to compare the carriage properties of ST618 and ST3081 and their relative propensity to cause invasive disease. We showed that during individual colonization events, ST3081 is carried at a higher density than ST618 during the first 7 days of carriage, but during cocolonization, the carriage density of ST618 is significantly increased in the first 24 hours to match ST3081 level; this is a transitory event, however, because during the next 6 days CFU counts are once again higher for ST3081 than for ST618. In line with this finding, results from our mouse invasive pneumonia model clearly demonstrate that the newly emerging ST3081 is significantly more virulent (and invasive) than the preexisting ST618 clone (Figures 2 and 3).

Serotype 1 isolates can be classified into 4 lineages depending on their geographic place of isolation [24, 41]. This geographic clustering may be due to the short carriage duration of serotype 1 strains, resulting in reduced geographic spread of the organism [42]. All STs identified in our study belong to the ST217 hypervirulent clonal complex responsible for epidemic outbreaks across the African meningitis belt [18, 20]. ST3081 is a single-locus variant of ST217 and quadruple-locus variant of ST618. To assess whether genetic recombination or point mutations explain the emergence of ST3081 [43, 44], we used whole-genome sequencing to determine the genomic differences between these 2 STs. Our results showed that ST3081 isolates exhibited lower sequence diversity than ST618 isolates, most likely a consequence of its recent emergence (in about 2002).The lower diversity in the ST3081 may be a consequence of a transmission bottleneck, which subsequently resulted in a lower population size, rather than the effect of negative selection. Further analysis of the distribution of SNPs in the 2 STs suggests that the population size of ST3081 is smaller than that of ST618 but is slowly expanding over time.

We also found that the *murF* gene, which is involved in the biosynthesis of peptidoglycan, is present in ST3081 isolates but absent in ST618 isolates. This is a significant finding, because impairment of this gene has previously been associated with inhibited growth and autolysis at certain environmental conditions, such as high temperatures and low environmental carbon dioxide levels, suggesting that ST3081 might be better adapted at surviving at high temperature conditions than ST618 and may therefore have a fitness advantage over ST618 [36]. This ST-specific gene, together with other uncharacterized genes, may contribute to differences in virulence between the STs. 

In addition, we found a single amino acid change in the pneumolysin toxin in ST3081 compared with ST618; this particular amino acid change has previously been associated with increased hemolysis in serotypes causing outbreaks [26]. Indeed, we showed that ST3081 has higher hemolytic activity than ST618 in vitro. Multiple studies have shown the importance of pneumolysin and its hemolytic activity in the virulence of the pneumococcus. Pneumolysin-deficient pneumococci have significantly lower ability to colonize the nasopharynx of mice, leading to reduced colonization density and shorter duration of colonization; they are also less able to cause invasive disease in mice, leading to prolonged survival compared with that in mice infected with pneumolysin-producing pneumococci [45–49]. The pneumolysin hemolytic activity–increasing amino acid change observed in ST3081 would hence increase virulence and tissue invasiveness, potentially allowing this ST to become the dominant ST in Gambia.

In the current study, we provided in vivo and genomic evidence that, within a given serotype, clonal properties may vary significantly and are an important determinant in the ability of pneumococcal clones to compete with each other, colonize the nasopharynx, and cause invasive disease. Our study highlights the importance of continuing surveillance to monitor the emergence of potentially more virulent and invasive pneumococcal clones and provides evidence addressing the intriguing question of whether clonal replacement is the result of naturally occurring competitive interactions within the host or the consequence of environmental and host influences.

## Supplementary Data

Supplementary materials are available at *The Journal of Infectious Diseases* online. Consisting of data provided by the authors to benefit the reader, the posted materials are not copyedited and are the sole responsibility of the authors, so questions or comments should be addressed to the corresponding author.

## Supplementary Material

Supplementary_Figure1

Supplementary_Table2

Supplementary_Table3

Supplementary_Table1

Supplementary_Figure_Legend

## Notes

***Acknowledgments.*** We thank all the individuals who participated in the study. We also acknowledge the use of the core sequencing facility at MRC Unit of The Gambia and the *S. pneumoniae* MLST database, which is housed at Imperial College, London.

***Author contributions.*** L. B. M. performed and analyzed the in vivo experiments and the hemolytic assays. C. E. provided and analyzed the epidemiological data. C. C., J. C., and D. E. performed and analyzed the genomic data. L. B. M., C. E., C. C., M. A., and A. K. wrote the manuscript. All authors discussed the results and commented on the manuscript. L. B. M. and C. E. are joint first authors, and M. A. and A. K. joint senior authors. M. A. and A. K. conceived and designed the experiments and supervised the study.

***Financial support.*** This work was supported by core funding from the University of Liverpool and the MRC and a research grant from the MRC.

***Potential conflicts of interest.*** All authors: No reported conflicts. All authors have submitted the ICMJE Form for Disclosure of Potential Conflicts of Interest. Conflicts that the editors consider relevant to the content of the manuscript have been disclosed.
