# Supplementary figures and images for: Comparative Genomic Analysis and In Vivo Modeling of Streptococcus pneumoniae ST3081 and ST618 Isolates Reveal Key Genetic and Phenotypic Differences Contributing to Clonal Replacement of Serotype 1 in The Gambia
Source: J Infect Dis. 2017 Sep 14;216(10):1318–27. doi: 10.1093/infdis/jix472 (PMC5853340; doi:10.1093/infdis/jix472)

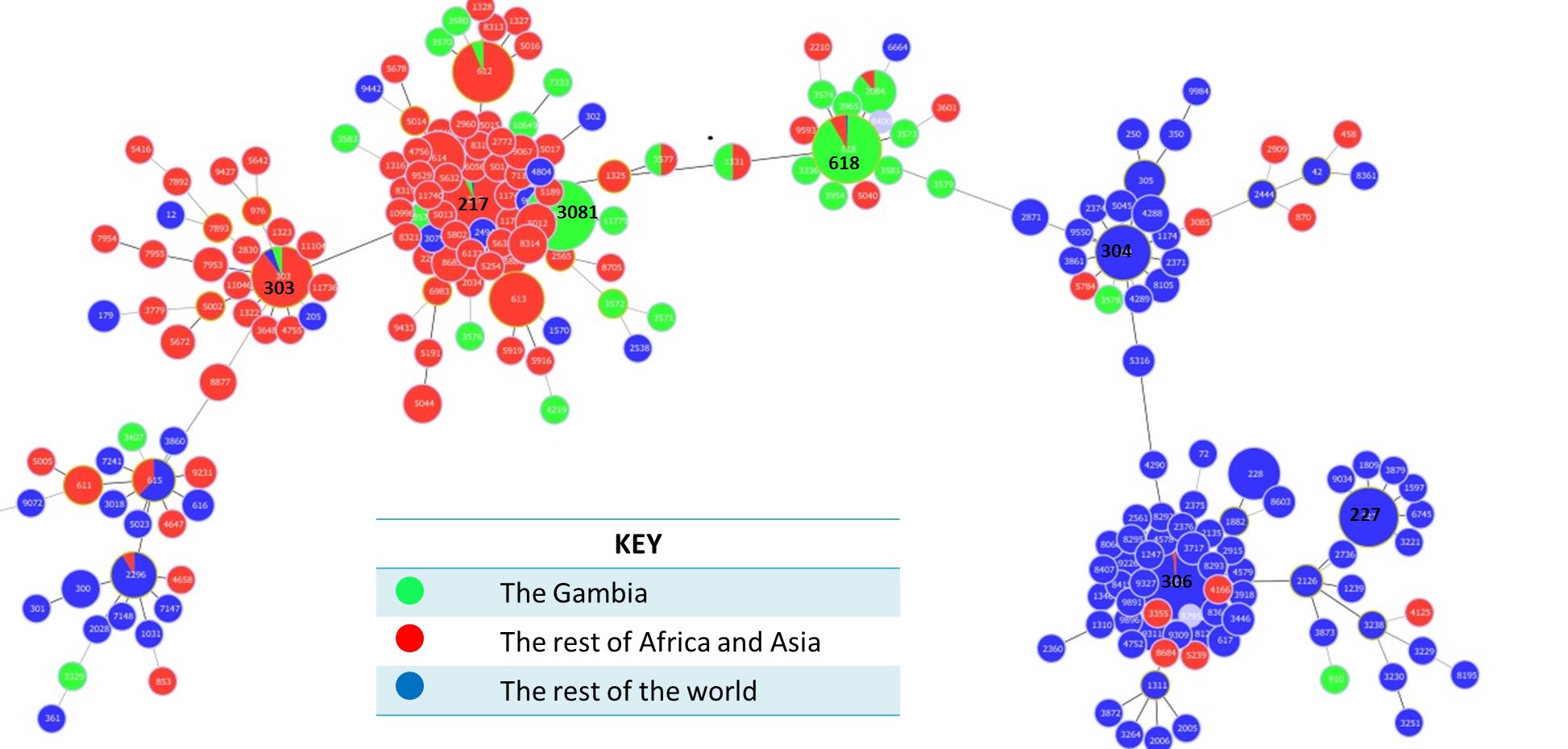

Supplement: Supplementary_Figure1 [file jix472_suppl_supplementary_figure1.jpeg]
