# Supplementary material for: Comparative Genomic Analysis and In Vivo Modeling of Streptococcus pneumoniae ST3081 and ST618 Isolates Reveal Key Genetic and Phenotypic Differences Contributing to Clonal Replacement of Serotype 1 in The Gambia
Source: J Infect Dis. 2017 Sep 14;216(10):1318–27. doi: 10.1093/infdis/jix472 (PMC5853340; doi:10.1093/infdis/jix472)
Supplement: Supplementary_Table2 [file jix472_suppl_supplementary_table2.docx]

**Supplementary Table 2: List of isolates used for the whole genome analysis.**

| **Sample ID** | **Sequence Type (ST)** | **ENA Accession Number** | **Year** |
| --- | --- | --- | --- |
| 6309_7#1 | ST618 | ERS024640 | 2005 |
| 6309_7#2 | ST618 | ERS024641 | 2005 |
| 6309_7#4 | ST 618 | ERS024643 | 2006 |
| 6308_8#17 | ST618 | ERS024704 | 2007 |
| 6308_8#18 | ST618 | ERS024732 | 2007 |
| 6308_8#7 | ST618 | ERS024694 | 2007 |
| 6309_7#13 | ST618 | ERS024652 | 2007 |
| 10702_4#1 | ST618 | ERS255551 | 1997 |
| 10702_4#10 | ST618 | ERS255548 | 1998 |
| 10702_4#12 | ST618 | ERS255549 | 1998 |
| 10702_4#13 | ST618 | ERS255565 | 1997 |
| 10702_4#14 | ST618 | ERS255545 | 1996 |
| 10702_4#15 | ST618 | ERS255555 | 1997 |
| 10702_4#16 | ST618 | ERS255569 | 1997 |
| 10702_4#17 | ST618 | ERS255552 | 1996 |
| 10702_4#18 | ST618 | ERS255550 | 2002 |
| 10702_4#2 | ST618 | ERS255571 | 1998 |
| 10702_4#20 | ST618 | ERS255553 | 1999 |
| 10702_4#21 | ST618 | ERS255559 | 1996 |
| 10702_4#22 | ST618 | ERS255574 | 1997 |
| 10702_4#23 | ST618 | ERS255572 | 1997 |
| 10702_4#24 | ST618 | ERS255562 | 1998 |
| 10702_4#25 | ST618 | ERS255564 | 1997 |
| 10702_4#26 | ST618 | ERS255547 | 1998 |
| 10702_4#27 | ST618 | ERS255578 | 1997 |
| 10702_4#28 | ST618 | ERS255570 | 1996 |
| 10702_4#29 | ST618 | ERS255563 | 1996 |
| 10702_4#3 | ST618 | ERS255554 | 2002 |
| 10702_4#30 | ST618 | ERS255575 | 1997 |
| 10702_4#31 | ST618 | ERS255573 | 1997 |
| 10702_4#32 | ST618 | ERS255576 | 1997 |
| 10702_4#34 | ST618 | ERS255577 | 1997 |
| 10702_4#4 | ST618 | ERS255546 | 1997 |
| 10702_4#5 | ST618 | ERS255558 | 1997 |
| 10702_4#7 | ST618 | ERS255566 | 1999 |
| 10702_4#8 | ST618 | ERS255568 | 1997 |
| 6308_8#15 | ST3081 | ERS024741 | 2007 |
| 6308_8#16 | ST3081 | ERS024703 | 2007 |
| 6308_8#4 | ST3081 | ERS024691 | 2007 |
| 6308_8#5 | ST3081 | ERS024692 | 2007 |
| 6308_8#6 | ST3081 | ERS024744 | 2007 |
| 6308_8#8 | ST3081 | ERS024695 | 2007 |
| 6308_8#9 | ST3081 | ERS024729 | 2007 |
| 6309_7#11 | ST3081 | ERS024650 | 2007 |
| 6309_7#14 | ST3081 | ERS024653 | 2007 |
| 6309_7#21 | ST3081 | ERS024717 | 2007 |
| 6309_7#5 | ST3081 | ERS024644 | 2007 |
| 6309_7#6 | ST3081 | ERS024712 | 2007 |
| 6309_7#7 | ST3081 | ERS024646 | 2007 |
| 6309_7#8 | ST3081 | ERS024647 | 2007 |
| 6309_7#9 | ST3081 | ERS024736 | 2007 |
| 6308_7#2 | ST3081 | ERS024665 | 2008 |
| 6308_7#3 | ST3081 | ERS024735 | 2008 |
| 6308_7#5 | ST3081 | ERS024668 | 2008 |
| 6308_8#10 | ST3081 | ERS024697 | 2008 |
| 6308_8#12 | ST3081 | ERS024730 | 2008 |
| 6308_8#13 | ST3081 | ERS024700 | 2008 |
| 6308_8#14 | ST3081 | ERS024701 | 2008 |
| 6308_8#19 | ST3081 | ERS024706 | 2008 |
| 6308_8#20 | ST3081 | ERS024707 | 2008 |
| 6308_8#21 | ST3081 | ERS024733 | 2008 |
| 6308_8#22 | ST3081 | ERS024709 | 2008 |
| 6309_7#15 | ST3081 | ERS024715 | 2008 |
| 6309_7#16 | ST3081 | ERS024655 | 2008 |
| 6309_7#17 | ST3081 | ERS024656 | 2008 |
| 6309_7#18 | ST3081 | ERS024742 | 2008 |
| 6309_7#23 | ST3081 | ERS024662 | 2008 |
| 6309_7#24 | ST3081 | ERS024718 | 2008 |
| 6308_7#10 | ST3081 | ERS024673 | 2009 |
| 6308_7#11 | ST3081 | ERS024674 | 2009 |
| 6308_7#12 | ST3081 | ERS024738 | 2009 |
| 6308_7#13 | ST3081 | ERS024676 | 2009 |
| 6308_7#8 | ST3081 | ERS024671 | 2009 |
| 6308_7#9 | ST3081 | ERS024721 | 2009 |
