# Supplementary material for: Comparative Genomic Analysis and In Vivo Modeling of Streptococcus pneumoniae ST3081 and ST618 Isolates Reveal Key Genetic and Phenotypic Differences Contributing to Clonal Replacement of Serotype 1 in The Gambia
Source: J Infect Dis. 2017 Sep 14;216(10):1318–27. doi: 10.1093/infdis/jix472 (PMC5853340; doi:10.1093/infdis/jix472)
Supplement: Supplementary_Table3 [file jix472_suppl_supplementary_table3.docx]

**Supplementary Table 3. Haemolytic activity of 14 isolates from ST618 and ST3081 isolates shown as OD_540_ of 85µg of total protein_._** **P-value<0.01 when analysed using a student t-test.

|  | **ST618** | **ST3081** |
| --- | --- | --- |
|  | 0.650193 | 0.72822 |
|  | 0.664268 | 0.698678 |
|  | 0.617486 | 0.7742 |
|  | 0.55842 | 0.751431 |
|  | 0.572129 | 0.636551 |
|  | 0.542694 | 0.580488 |
|  | 0.422872 | 0.630519 |
| **Average**** | **0.575437** | **0.685727** |
| **SEM** | **0.075495** | **0.066181** |
