# Supplementary material for: Comparative Genomic Analysis and In Vivo Modeling of Streptococcus pneumoniae ST3081 and ST618 Isolates Reveal Key Genetic and Phenotypic Differences Contributing to Clonal Replacement of Serotype 1 in The Gambia
Source: J Infect Dis. 2017 Sep 14;216(10):1318–27. doi: 10.1093/infdis/jix472 (PMC5853340; doi:10.1093/infdis/jix472)
Supplement: Supplementary_Table1 [file jix472_suppl_supplementary_table1.docx]

**Supplementary Table 1. *S. pneumoniae* isolates used in this study.**

| **Year of Study** | **Geographic area*** | **Number of serotype 1 isolates (%)** | **Reference** |
| --- | --- | --- | --- |
| **Invasive isolates** |  |  |  |
| 1995 - 2003 | WR | 93 (73.2) | [[13](#_ENREF_13)] |
| 1995 - 2003 | URR & CRR | 34 (26.8) | [[13](#_ENREF_13)] |
| Total |  | 127 (100) |  |
| 2004 - 2014 | WR | 45 (40) | This study |
| 2004 - 2014 | URR & CRR | 145 (60) | This study |
| Total |  | 190 (100) |  |
| **Carriage isolates** |  |  |  |
| 2003 - 2009 | WR | 21 (100) | [22] |

*WR; Western Region (urban), CRR; Central River Region (rural), URR; Upper River Region (rural)
