# Supplementary material for: Comparative Genomic Analysis and In Vivo Modeling of Streptococcus pneumoniae ST3081 and ST618 Isolates Reveal Key Genetic and Phenotypic Differences Contributing to Clonal Replacement of Serotype 1 in The Gambia
Source: J Infect Dis. 2017 Sep 14;216(10):1318–27. doi: 10.1093/infdis/jix472 (PMC5853340; doi:10.1093/infdis/jix472)
Supplement: Supplementary_Figure_Legend [file jix472_suppl_supplementary_figure_legend.docx]

## Supplementary Figure Legend

**Supplementary Figure 1: geoBURST analysis comparing Gambian pneumococcal serotype 1 isolates to the rest of Africa, Asia and globally.**

Each circle represents a single sequence type (ST). The area of each circle is proportional to the number of isolates included in the analysis.
